# Characterization of Browning, Chlorogenic Acid Content, and Polyphenol Oxidase Activity in Different Varietal Types of Eggplant (*Solanum melongena*) for Improving Visual and Nutritional Quality

**DOI:** 10.3390/plants13081059

**Published:** 2024-04-09

**Authors:** Gloria Villanueva, Santiago Vilanova, Mariola Plazas

**Affiliations:** Instituto de Conservación y Mejora de la Agrodiversidad Valenciana, Universitat Politècnica de València, Camino de Vera 14, 46022 Valencia, Spain; sanvina@upvnet.upv.es (S.V.); maplaav@btc.upv.es (M.P.)

**Keywords:** *Solanum melongena*, chlorogenic acid, polyphenol oxidase, browning, varietal type

## Abstract

Eggplant (*Solanum melongena* L.) breeding for fruit quality has mostly focused on visual traits and nutritional and bioactive compounds, including chlorogenic acid. However, higher contents of chlorogenic acid may lead to more pronounced fruit flesh browning. We examined a diverse collection of 59 eggplant accessions across five varietal types (‘black oval’, ‘striped’, ‘anthocyanin-free’, ‘purple’, and ‘black elongated’) to evaluate the degree of browning, polyphenol oxidase (PPO) activity, and chlorogenic acid (CGA) content. The results reveal moderate correlations among these traits, with no clear differences among the varietal types, suggesting that other factors, including genetic variation, might significantly influence these traits. Notably, ‘black oval’ accessions demonstrated higher browning and PPO activity, whereas ‘striped’ accessions showed low variability. The identification of genotypes with lower browning and higher CGA content highlights opportunities for targeted genotype selection to improve eggplant chlorogenic acid content while maintaining low or moderate browning, pointing towards the importance of genetic considerations in breeding strategies to reduce browning and enhance nutritional value.

## 1. Introduction

Eggplant (*Solanum melongena* L.), also known as brinjal or aubergine, is widely cultivated worldwide, being established as the fifth highest-yielding vegetable crop [1]. Eggplant exhibits a wide diversity of phenotypic, physiological, and biochemical traits encompassing a range of growth habits and vegetative traits, along with a diversity of fruit shapes, sizes, and colors [2,3,4]. Over the last two decades, eggplant yields have almost doubled, reflecting significant advancements in agricultural practices and varietal improvements. Consequently, alongside these advancements, the enhancement of visual and nutritional qualities has become a key focus in plant breeding, aligning with consumer demands and health trends [1,5].

Nutritionally, eggplant fruits constitute a valuable source of essential nutrients, including vitamins, minerals, and proteins, along with bioactive compounds like phenolic acids and flavonoids, which offer several health benefits [6]. Chlorogenic acid (5-O-caffeoyl-quinic acid; CGA) is the predominant phenolic acid in eggplant [7,8], noted for its potential health-promoting properties, including antioxidant, anti-carcinogenic, anti-inflammatory, analgesic, antibacterial, cardioprotective, and anti-diabetic properties [9,10]. However, the presence of CGA and other phenolic compounds plays a critical role in the postharvest quality of eggplant due to their susceptibility to enzymatic browning. The disruption of cell structures allows phenolic compounds, mostly contained within vacuoles, to interact with polyphenol oxidases (PPOs), enzymes located in chloroplasts. This interaction initiates the PPO-catalyzed oxidation of phenolics to quinones, which further react non-enzymatically with oxygen and other compounds, such as sulfhydryl groups and proteins, forming brown-colored pigments. Enzymatic browning, influenced by PPO activity and phenolic levels, notably compromises the visual and nutritional value of produce, presenting a significant challenge in maintaining quality during postharvest processing and storage in the food industry [11].

While considerable research has focused on PPOs due to the economic implications of enzymatic browning, their role in plant physiology has been less explored. It is understood that PPOs may play a part in signal transduction and defense mechanisms throughout plant growth, potentially correlating with biotic stress resistance [12,13]. The roles of PPOs in plant defense are believed to include a range of processes, from the potential toxicity of quinones to the strategic sequestration of proteins, the creation of physical barriers through quinone–protein interactions, and involvement in reactive oxygen species (ROS) signaling, all of which contribute to the plant’s overall defense strategy [14].

The main objective of this study is to evaluate the genetic diversity and quality traits across a diverse collection of 59 eggplant (*S. melongena*) accessions, categorized into five distinct varietal types: ‘black oval’, ‘striped’, ‘anthocyanin-free’, ‘purple’, and ‘black elongated’. Our analysis focuses on the variability, correlations, and trends in visual and nutritional traits, such as browning, polyphenol oxidase (PPO) activity, and chlorogenic acid (CGA) content. This research aims to provide critical insights for the selection and breeding of eggplant varieties, with a particular focus on enhancing key quality attributes.

## 2. Materials and Methods

### 2.1. Plant Material and Cultivation Conditions

**Figure 1:**
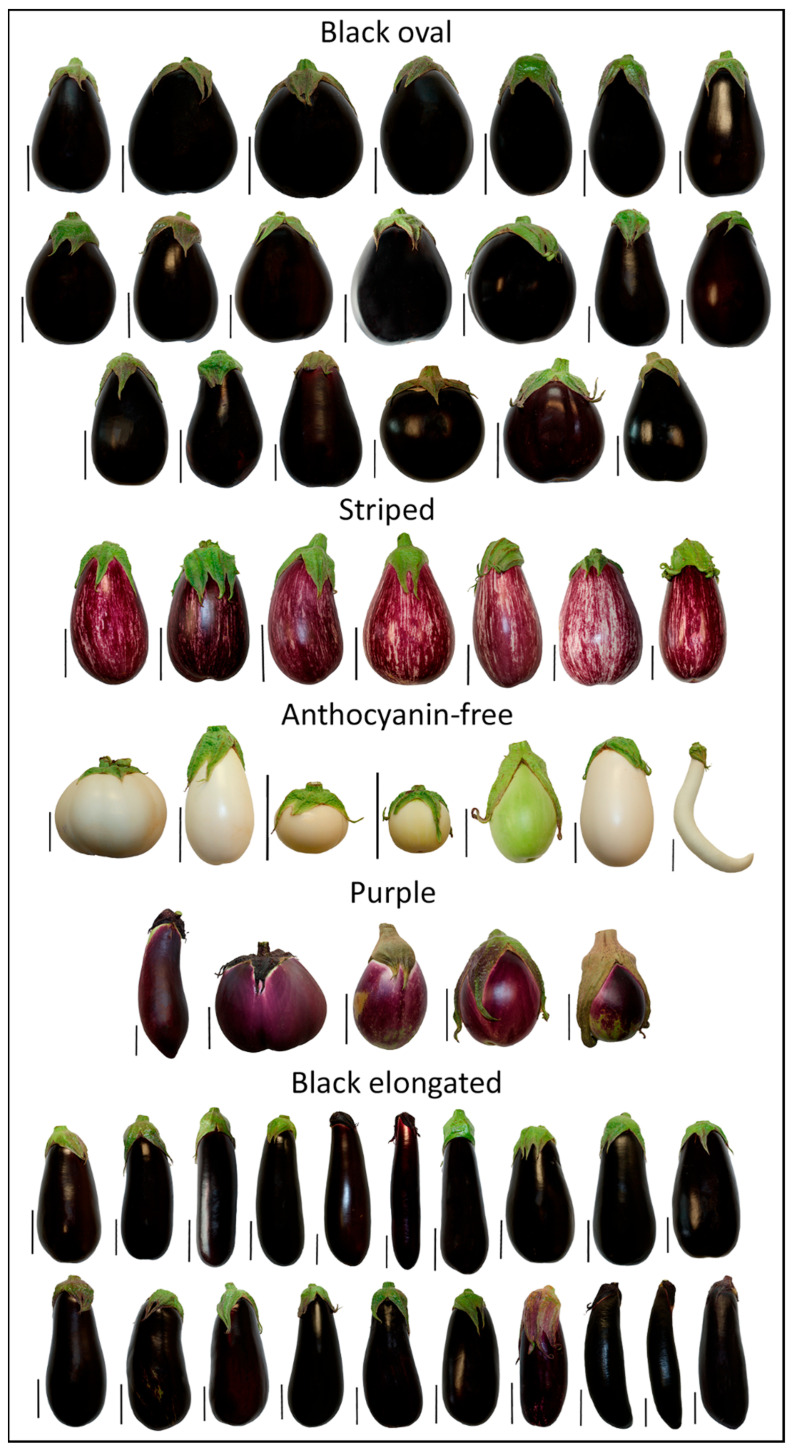
Collection of 59 eggplant (*S. melongena*) accessions used in the study, categorized into five varietal types: ‘black oval’, ‘striped’, ‘anthocyanin-free’, ‘purple’, and ‘black elongated’. Fruits are aligned with a black line scale of 5 cm for size reference.

This study involved a collection of 59 accessions of eggplant (*Solanum melongena*), categorized into five varietal types: ‘black oval’, ‘striped’, ‘anthocyanin-free’, ‘purple’, and ‘black elongated’ eggplants (Figure 1). Eggplants were first categorized by color and then, specifically within the ‘black’ variety, further classified into ‘oval’ and ‘elongated’ based on shape. These accessions were cultivated in open field conditions located on the campus of the Universitat Politècnica de València (39°28′55″ N, 0°20′11″ W, 7 m above sea level) during the summer season (June to October 2018), with the plants distributed in a planting frame design consisting of 180 cm between rows and 60 cm between plants within each row. Three plants of each accession were grown under these conditions, with a drip irrigation system employed for consistent irrigation and fertilization.

### 2.2. Fruit Collection and Browning Measurement

For the total collection of eggplant accessions, up to 15 self-pollinated fruits were harvested from plants of each accession at the commercial stage, approximately 20 days post-anthesis (20 DPA) for subsequent analysis.

The evaluation of browning in the flesh of the eggplant fruits was performed using a Minolta CR-300 Chroma Meter colorimeter (Minolta Co., Ltd., Osaka, Japan), utilizing the CIELAB color space system (L*, a*, b*) [15]. Three distinct measurements were taken from the flesh of each fruit immediately after cutting (0 min) and after 10 min. The color difference (CD) was determined by calculating the Euclidean distance between both measurements:

$$CD=\sqrt{{\left(L_{10min}-L_{0min}\right)}^{2}+{\left(a_{10min}-a_{0min}\right)}^{2}+{\left(b_{10min}-b_{0min}\right)}^{2}}$$

### 2.3. Fruit Processing and Composition Analyses

After the browning measurements, fruits were cut, peeled, and immediately frozen in liquid nitrogen, then stored at −80 °C. These samples were subsequently freeze-dried and ground into powder. For each accession, three random composite samples, consisting of material from up to five fruits per accession, were prepared, with an average color difference (CD) calculated for each.

Total protein content was determined from 50 mg of freeze-dried powdered material, a crucial step for establishing the activity of polyphenol oxidase (PPO) relative to protein concentration. The extraction buffer employed included sodium phosphate buffer (pH 6; 0.1 M), supplemented with 1% polyvinylpyrrolidone (PVP), 2% polyvinylpolypyrrolidone (PVPP), 1% Triton X-100 (Sigma-Aldrich, St. Louis, MO, USA), and 30 mM ascorbic acid (Panreac Química, Barcelona, Spain) to mitigate rapid phenol oxidation. The Bradford method [16] was utilized to quantify the extracted total protein content, with absorbance readings obtained at 595 nm using a 96-well Microplate Reader iMark (BioRad, Hercules, CA, USA). Analyses were performed in triplicate on composite fruit samples to ensure accuracy and reliability.

The activity of polyphenol oxidase (PPO) in these protein extracts was evaluated by adding a sodium phosphate buffer (pH 6; 0.1 M), containing both disodium phosphate (Na_2_HPO_4_) and monosodium phosphate (NaH_2_PO_4_) (Panreac Química, Spain), and a chlorogenic acid (CGA) substrate (Sigma-Aldrich, USA) at a precise concentration. The kinetic activity was determined by measuring the increase in absorbance at 405 nm [10,17] using the same Microplate Reader. The measurement of PPO activity was also performed in triplicate using the same stock of lyophilized material. An enzymatic activity unit was defined as the amount of enzyme causing a change in absorbance of 0.1 OD/min. PPO activity was expressed as units per milligram of protein (U/mg protein).

Chlorogenic acid (CGA) was extracted via an ultrasonic bath using 0.1 g of freeze-dried powdered material, as described by Helmja et al. [18]. The subsequent analysis of CGA content was conducted using high-performance liquid chromatography (HPLC) with a 1220 Infinity LC System (Agilent Technologies, Santa Clara, CA, USA) equipped with a binary pump, an automatic injector, and a UV detector, following the methodologies reported [19,20]. Extractions from both individual fruits and mixed samples were performed, with each analysis conducted in triplicate.

### 2.4. Statistical Analysis

Means and ranges were calculated for each trait within the different varietal types. The Shapiro–Wilk test was applied to assess the normality of the data for each trait. In cases of normal distribution, an analysis of variance (ANOVA) was conducted to determine significant differences in means across varietal types. For non-normally distributed data, a non-parametric Kruskal–Wallis test was performed. Post-hoc analysis for significant differences was carried out using the Bonferroni correction method, with a significance threshold set at *p* < 0.05. All statistical analyses were performed using Statgraphics Centurion XIX software (Statgraphics Technologies, Inc., The Plains, VA, USA).

Pearson pairwise correlation coefficients were calculated among traits using the R package GGally [21]. Histograms with density curves and scatter plots were drawn using the R packages ggplot2 [22] and RColorConesa [23].

## 3. Results

### 3.1. Distribution Analysis and Characterization of Varietal Types

**Figure 2:**
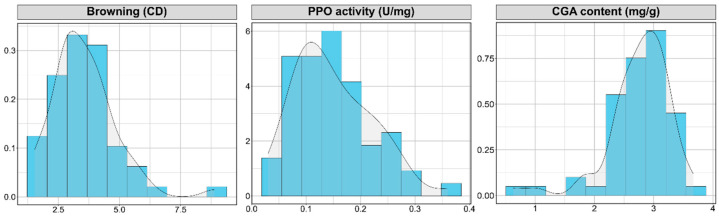
Histograms with density curves of browning (CD), PPO activity (U/mg), and CGA content (mg/g) across a collection of 59 eggplant (*S. melongena*) accessions used in the study.

The distribution of polyphenol oxidase (PPO) activity did not differ significantly from a normal distribution, as shown by the histogram and density curve. In contrast, browning exhibited a right-skewed tail, while chlorogenic acid (CGA) content was skewed towards the left, indicating non-normal distributions (Figure 2).

Significant differences were observed in browning and polyphenol oxidase (PPO) activity among different varietal types, while chlorogenic acid (CGA) content did not exhibit significant differences (Table 1). Particularly, the ‘black oval’ group demonstrated a significantly higher degree of browning compared to the ‘black elongated’ group, while the other varietal types did not differ significantly among them in this trait. Additionally, the ‘black oval’ group displayed higher mean PPO activity compared to the accessions of ‘striped’ and ‘anthocyanin-free’ groups, while this variation was not observed for ‘purple’ and ‘black elongated’ groups (Table 1).

Across all varietal types, wide ranges of variation were observed for browning, PPO activity, and CGA content. Specifically, the ‘striped’ varietal type accessions had the lowest ranges of these traits, with differences among accession means of 1.8-fold for browning, 2.5-fold for PPO activity, and 1.2-fold for CGA content. Conversely, the highest ranges were exhibited by the ‘purple’ accessions for browning (5.8-fold), the ‘black oval’ for PPO activity (3.3-fold), and the ‘anthocyanin-free’ accessions for CGA content (5.1-fold) (Table 1).

### 3.2. Trends in Trait Associations across Varietal Types

**Figure 3:**
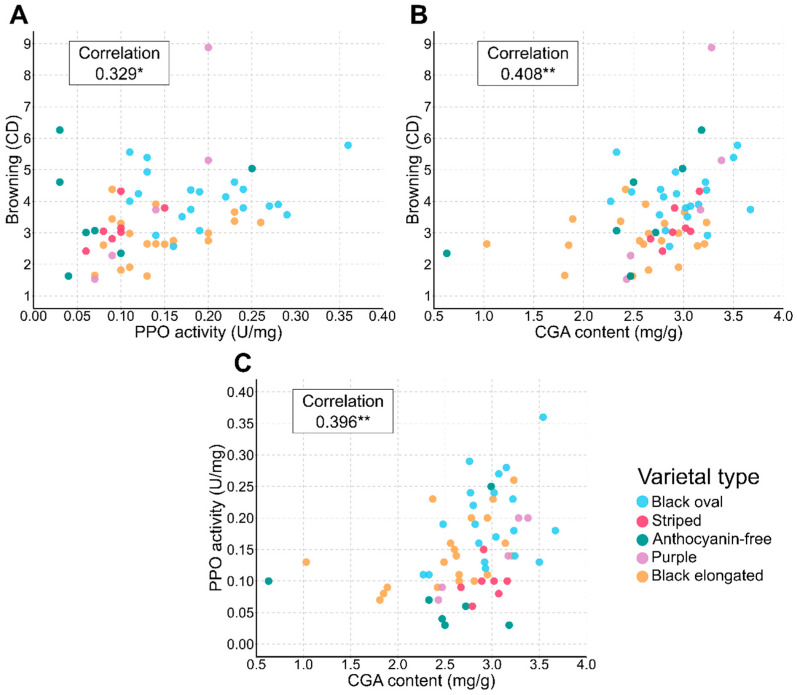
Scatter plots of mean values with Pearson correlations of browning (CD) and PPO activity (U/mg) (**A**), browning (CD) and CGA content (mg/g) (**B**), and PPO activity (U/mg) and CGA content (mg/g) (**C**). Each dot represents an individual accession, color-coded by varietal type: ‘black oval’, ‘striped’, ‘anthocyanin-free’, ‘purple’, and ‘black elongated’. Significant pairwise trait correlations are marked accordingly (“*” *p*-value < 0.05, “**” *p*-value < 0.01).

A general trend of positive correlations was observed across all trait comparisons, with statistically significant associations revealed among the studied traits (Figure 3). Browning exhibited a moderate positive correlation with PPO activity (r = 0.329, *p* < 0.05) (Figure 3A) and a higher positive correlation with CGA content (r = 0.408, *p* < 0.01) (Figure 3B). Additionally, a notable significant positive correlation was detected between PPO activity and CGA content (r = 0.396, *p* < 0.01) (Figure 3C).

‘Black oval’ accessions predominantly exhibited higher browning and PPO activity values, whereas the ‘black elongated’ group generally showed lower values of these traits (Figure 3A); both varietal types displayed mid-range CGA contents (Figure 3B,C). ‘Anthocyanin-free’ accessions were predominantly characterized by lower PPO activity (Figure 3A,C) and exhibited a wide range in both browning and CGA content (Figure 3B,C). ‘Purple’ accessions demonstrated variability in all traits, suggesting no consistent trend. In contrast, the ‘striped’ accessions were characterized by a relative clustering, associated with a higher CGA content, and moderate to low values for browning and PPO activity, respectively (Figure 3).

## 4. Discussion

Alongside advances in crop yield, the importance of enhancing nutritional and visual quality in fruit crops has gained increasing recognition [24]. Notably, significant progress has been made in understanding the biosynthesis pathways of phenolic acids and identifying the genes and QTLs involved in the Solanaceae family [25,26,27,28]. In eggplant, enzymatic browning, which results from the oxidation of phenolics released from vacuoles by polyphenol oxidases (PPOs), leads to a reduction in fruit quality. Previous studies have established strong correlations among browning, phenolic content, and PPO activity [11,29]; however, our research aligns more closely with studies reporting moderate positive correlations, underscoring the complex interplay affecting eggplant quality and highlighting variability among different eggplant cultivars [10,20,30,31].

Among the phenolic compounds in eggplants, anthocyanins, a specific subgroup of flavonoids, are the natural pigments found in the fruit peel [6]. This distinctive coloration has served as a criterion for the varietal classification of eggplant cultivars in this study. Consequently, eggplants have been categorized into five varietal types, including ‘striped’, ‘anthocyanin-free’, ‘purple’, and ‘black oval’ or ‘black elongated’, with the latter differentiated by their distinct shapes. While previous research has reported broad variability in traits such as browning, PPO activity, and chlorogenic acid (CGA) content across both wild and cultivated species related to *S. melongena* [10,11,20,29,32], analyses focusing on varietal types within *S. melongena* have been limited. Our findings reveal that, in general terms, no clear distinctions in browning, PPO activity, and CGA content were observed among the varietal types, with each showing a wide range of variation. This may suggest that the differentiation in these traits may not be directly linked to distinct peel pigmentation but rather depends on the individual genotype. Despite this overall trend, specific differences were observed, such as increased browning in the ‘black oval’ group compared to the ‘black elongated’ group and higher PPO activity in the ‘black oval’ group relative to both ‘striped’ and ‘anthocyanin-free’ groups. This could suggest that the ‘black oval’ varietal type tends to show elevated levels of both browning and PPO activity. Interestingly, the ‘striped’ accessions displayed a relative clustering, indicating less variability within this varietal type compared to others. Furthermore, varietal types with lower or no presence of anthocyanins appear to display reduced PPO activity. The general variability observed within varietal types could facilitate the selection of accessions with desirable quality characteristics, such as reduced browning and increased chlorogenic acid levels, offering potential pathways for breeding programs aimed at improving eggplant quality traits.

This study underscores the significant roles of PPO activity and CGA content in eggplant fruit flesh browning, yet the complexity of this trait suggests the involvement of additional factors. Non-enzymatic browning reactions, particularly the Maillard reaction, have been reported as notable contributors [33,34]. Furthermore, some studies have reported the important roles of other antioxidants, various enzymes, and environmental factors such as temperature and pH, along with specific storage conditions, as influencing factors in the browning of eggplants [35,36,37,38].

## 5. Conclusions

This research highlights the complexity and variability of browning, polyphenol oxidase (PPO) activity, and chlorogenic acid (CGA) content in eggplant fruits. The identification of moderate correlations among these traits suggests the potential influence of additional factors on fruit quality. The absence of clear differences in varietal types suggests that genotypic factors predominantly determine these traits. Notably, the identification of specific genotypes with low browning and high CGA content highlights the opportunity to select genotype selection to enhance postharvest quality in eggplants.

## Figures and Tables

**Table 1:** Mean values and range of browning (CD), PPO activity (U/mg), and CGA content (mg/g) of five *S. melongena* varietal types: ‘black oval’, ‘striped’, ‘anthocyanin-free’, ‘purple’, and ‘black elongated’. Different letters represent significant differences according to the Bonferroni multiple range test (*p*-value < 0.05).

Trait	Black Oval (n = 20)	Striped (n = 7)	Anthocyanin-Free (n = 7)	Purple (n = 5)	Black Elongated (n = 20)
Browning (CD)	4.13 b	3.22 ab	3.71 ab	4.34 ab	2.85 a
	(2.57–5.78)	(2.42–4.32)	(1.63–6.26)	(1.53–8.88)	(1.63–4.38)
PPO Activity (U/mg)	0.20 b	0.10 a	0.08 a	0.14 ab	0.14 ab
	(0.11–0.36)	(0.06–0.15)	(0.03–0.25)	(0.07–0.20)	(0.07–0.26)
CGA content (mg/g)	2.98	2.93	2.40	2.95	2.55
	(2.27–3.67)	(2.67–3.16)	(0.63–3.18)	(2.43–3.38)	(1.03–3.23)

## Data Availability

The data presented in this study are available on request from the authors. The raw data supporting the conclusions of this article will be made available by the authors on request.
